# Sex-related differences in symptom presentation of patients with aneurysmal subarachnoid hemorrhage

**DOI:** 10.12688/f1000research.124123.2

**Published:** 2023-03-13

**Authors:** Laura Philine Westphal, Stefan Yu Bögli, Jana Werner, Francesca Casagrande, Emanuela Keller, Giovanna Brandi

**Affiliations:** 1 Department of Neurology, University Hospital Zurich and University of Zurich, Zurich, 8091, Switzerland; 2 Neurocritical Care Unit, Institute of Intensive Care Medicine, University Hospital Zurich and University of Zurich, Zurich, 8091, Switzerland; 3 Department of Neurosurgery and Clinical Neuroscience Center, University Hospital Zurich and University of Zurich, Zurich, 8091, Switzerland

**Keywords:** aneurysmal subarachnoid hemorrhage, symptom presentation, gender medicine, sex-related differences, women

## Abstract

**Background: **In patients with myocardial infarction, atypical symptoms at onset have been demonstrated in women. We aimed to investigate the presence of sex-related differences in symptom presentation in patients with aneurysmal subarachnoid hemorrhage (aSAH) to enable earlier diagnosis and treatment.

**Methods:** We assessed symptoms on admission to hospital in 343 patients with aSAH in this retrospective single-center cohort-study. Univariate statistical analysis was performed by comparing sexes including the whole study population and subgroups (dichotomized using Fisher scale 1-2 vs. 3-4, WFNS grade 1-3 vs. 4-5, and anterior vs. posterior circulation aneurysms, respectively).

**Results:** The majority of patients was female (63.6%, n=218, vs. 36.4%, n=125), the mean age 57.4 years (standard deviation (SD) 13.3) with older women compared to men (59.2, SD 13.8, vs. 54.4, SD 11.6; p=0.003). Anterior communicating artery (AcomA) aneurysms were most common (30.9%, n=106), predominantly in men (43.2%, n=54, vs. 23.9%, n=52; p=0.0002), whereas posterior communicating artery (PcomA) aneurysms were more frequent in women (19.3%, n=42, vs. 8.8%, n=11; p=0.005). Exercise-induced headache was more often reported by men (10.4%, n=13, vs. 5%, n=11; p=0.04) in all patients as well as in the subgroup of WFNS 1-3. Anisocoria was more frequent in women within the subgroup of severely impaired consciousness (WFNS 4-5; 25.3%, n=22, vs. 10.7%, n=6; p=0.032). For all other symptoms, there was no evidence for sex-specific differences in the whole study group as well as in subgroups.

**Conclusion:** Our results show no evidence for relevant sex-related differences in symptom presentation at onset in aSAH patients. Women presenting with an acute onset anisocoria should be screened even more carefully for an underlying ruptured Pcom aneurysm.

## Introduction

Aneurysmal subarachnoid hemorrhage (aSAH) accounts for 5% of all strokes and for 75-80% of spontaneous SAH with an overall incidence of 6-8/100.000 people per year in Western populations. ^1^^,^^2^ As aSAH occurs at a younger age and has a high case fatality, the loss of productive life years ranges on the same level as the one from cerebral infarction. ^2^^,^^3^ Therefore, the assessment and accurate interpretation of clinical symptoms including the knowledge of possible sex-related differences in patients with aSAH is a key factor for rapid and correct diagnosis as well as further monitoring and treatment decisions.

In cardiovascular disease as myocardial infarction (MI), several studies demonstrated that chest pain is the most common symptom in males, while women more frequently suffered from atypical or unspecific associated symptoms such as dizziness, nausea, palpitations and pain or discomfort in the jaw, neck, arms or between the shoulder blades resulting in higher rates of unrecognized MI. ^4^^,^^5^ This sex-related difference in symptom presentation does not only contribute to lower rates of diagnosis of MI in women, but also to delayed or prevented treatment leading to worsened outcome in women compared to men. ^5^^–^^8^

Regarding sex-related differences in aSAH patients, it is known that aSAH affects more women than men ^2^^,^^9^ and women have a higher risk of death from SAH increasing with age. ^10^ Moreover, ruptured aneurysms in women are mainly found in the internal carotid artery (ICA), whereas in men in the anterior cerebral artery (ACA). ^11^ However, sex-related differences in symptom presentation have not been examined in-depth in this patient group so far.

We aimed to investigate if symptom presentation at onset in patients with aSAH differed between men and women after correction for possible confounders as aneurysm location and severity of hemorrhage. Sex-related atypical or different symptoms could thereby identify patients at higher risk for delayed diagnosis, which could lead to an increased focus on more diverse symptoms and the need for a more detailed anamnesis at symptom onset.

## Methods

### Study design and setting

**Figure 1.**
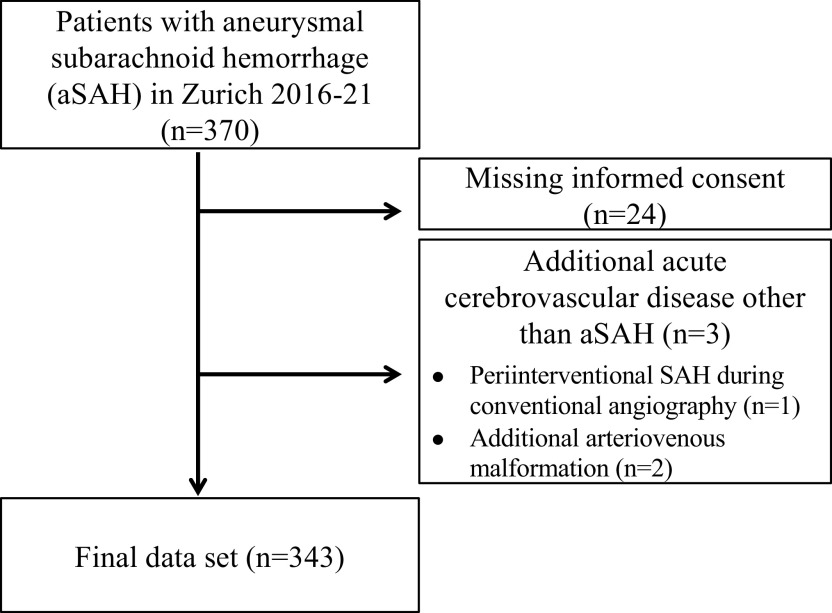
Flow chart of study population.

In this retrospective single-center cohort-study, we screened 370 and enrolled 343 consecutive patients with acute aSAH admitted to the Neurocritical Care Unit (NCCU) of the University Hospital Zurich, Switzerland, as a tertiary care center between January 2016 and December 2021. Patients were also included when they first presented in a primary or secondary care center with following transfer to the NCCU of the University Hospital Zurich. Patients were excluded when presenting with additional other acute cerebrovascular diseases (n=2) or confounding sources of bleeding (n=1) in order to provide a homogenous patient cohort as well as when a written informed consent was missing (n=24), see Figure 1 for a flow chart of the study population. aSAH was diagnosed by radiological findings on computed tomography (CT) and after evidence of a ruptured aneurysm on CT-angiography (CTA) scans on admission or subsequent digital substraction angiography (DSA). Patients with aSAH included intracranial aneurysms of the anterior and posterior circulation as well as all severity levels defined by the Fisher grading scale rating the risk of vasospasms based on the amount and distribution of blood on CT scans with localized blood clots or layers ≥1 mm in fissures or vertical cisterns indicating the highest risk of vasospasms. ^12^ Furthermore, all degrees of clinical impairment defined by the World Federation of Neurosurgical Societies (WFNS) scale using the Glasgow coma scale (GCS) for the level of consciousness combined with the presence or absence of focal deficits with higher grades indicating a worse patient condition were included. ^13^

Written informed consent was obtained in all patients included in the study or next of kin. The study was performed according to the ethical guidelines of the Canton of Zurich with approval of the local ethics committee of Zurich (KEK: 2022-00270).

### Clinical variables

On admission to hospital, we assessed demographic and clinical data, comorbidities presented by the Charlson comorbidity index (CCI) and radiologic findings as the aneurysm location, the presence of hydrocephalus defined as a relative bicaudate index > 1.6 on CT scan, intraventricular hemorrhage or additional intracerebral hemorrhage. ^14^^,^^15^ The severity of aSAH based on radiologic findings was quantified using the Fisher score, the decline of consciousness and severity of neurological impairment was graded by the WFNS grade. Initial GCS was defined as the first documented score by the emergency service, primary, secondary or tertiary care centers, whereas GCS on admission was the first documented score on admission to the University Hospital Zurich. Outcome was classified by the Glasgow Outcome Scale-Extended (GOS-E) offering a five-point scale with high levels indicating a good patient recovery ^16^ assessed by physicians of the department of neurosurgery at the University Hospital Zurich during follow-up at three months after onset of bleeding.

### Statistical analysis

Descriptive or categorical variables are reported as counts/percentages, continuous variables as mean ± standard deviation or as median including the interquartile range (IQR) as appropriate. For the analysis of associated factors, patient characteristics and presented symptoms of patients were dichotomized by sex. For subgroup analyses, patients dichotomized by sex were stratified by the Fisher scale, WFNS grade and aneurysm location, respectively. We tested all continuous data for normal distribution using the Shapiro-Wilk's test. Categorical variables were compared with Pearson's χ ^2^ or Fisher's exact test, continuous variables using the t-test or Mann–Whitney U test for parametric and non-parametric data, respectively, where appropriate. There were no missing values regarding the variables of clinical symptom presentation and there was no loss to follow-up at three months after onset of aSAH. P-values ≤0.05 were considered to be statistically significant. All calculations were performed using SPSS version 26.

## Results

### Patient data and baseline characteristics

We screened 370 patients with aSAH and included 343 into the analysis. A detailed flow chart of the patients included in the study is provided in Figure 1. For all patients a follow-up at three months after onset of aSAH could be assessed or was classified accordingly if patients died within the follow-up period. There were no missing data for the assessed clinical variables.

As presented in Table 1, the majority of patients was female (n=218, 63.6%). Women were older than men (59.2, SD 13.8, vs. 54.4, SD 11.6; p=0.003). Most aneurysms were located in the anterior circulation (79%, n=217) with the anterior communicating artery (AcomA) being the most common aneurysm location (30.9%, n=106) predominantly in men (43.2%, n=54, vs. 23.9%, n=52; p=0.0002), whereas aneurysms of the posterior communicating artery (PcomA) were more frequent in women (19.3%, n=42, vs. 8.8%, n=11; p=0.005). 70.5% (n=241) of all patients demonstrated an intraventricular hemorrhage (IVH) at onset with no evidence of a sex difference (p=0.81).

**Table 1.** Baseline characteristics of aSAH patients.

Characteristic	All (n=343)		Male (n=125)		Female (n=218)		*p*
**Demographic factors**							
Female sex, n (%)	218	(63.6)	n.a.	(n.a.)	218	(63.6)	n.a.
Age, mean (SD)	57.4	(13.3)	54.4	(11.6)	59.2	(13.8)	**<0.01**
CCI, median (IQR)	0	(0-2)	0	(0-2)	0	(0-1.3)	0.85
WLST, n (%)	39	(11.4)	11	(8.8)	28	(12.8)	0.29
GOS-E at 3 months, n (%)							
1	60	(17.8)	17	(13.8)	43	(20.0)	**<0.01**
2	12	(3.6)	3	(2.4)	9	(4.2)	
3	53	(15.7)	21	(17.1)	32	(14.9)	
4	33	(9.8)	7	(5.7)	26	(12.1)	
5	45	(13.3)	22	(17.9)	23	(10.7)	
6	39	(11.5)	22	(17.9)	17	(7.9)	
7	54	(16.0)	13	(10.6)	41	(19.1)	
8	42	(12.4)	18	(14.6)	24	(11.2)	
**Imaging**							
Aneurysm location, n (%)							
Anterior circulation	217	(79.0)	102	(81.6)	169	(77.5)	0.41
ICA	27	(7.9)	9	(7.2)	18	(8.3)	0.73
MCA	85	(24.8)	28	(22.4)	57	(26.1)	0.44
AComA	106	(30.9)	54	(43.2)	52	(23.9)	**<0.001**
ACA	6	(1.7)	1	(0.8)	5	(2.3)	0.31
VA	11	(3.2)	5	(4)	6	(2.8)	0.53
PCA	4	(1.2)	1	(0.8)	3	(1.4)	0.63
PComA	53	(15.5)	11	(8.8)	42	(19.3)	**<0.01**
BA	18	(5.2)	6	(4.8)	12	(5.5)	0.78
PICA	18	(5.2)	5	(4)	13	(6.0)	0.43
Fisher scale, n (%)							
1	13	(3.8)	4	(3.2)	9	(4.1)	0.86
2	24	(7.0)	8	(6.4)	16	(7.4)	
3	143	(41.8)	56	(44.8)	87	(40.1)	
4	162	(47.4)	57	(45.6)	105	(48.4)	
ICH	95	(27.7)	37	(29.6)	58	(26.6)	0.62
SDH	33	(9.6)	17	(13.6)	16	(7.3)	0.09
Hydrocephalus	179	(52.2)	65	(52)	114	(52.3)	1
IVH	241	(70.5)	87	(69.6)	154	(71)	0.81
Basal cisterns involved	218	(63.7)	82	(65.6)	136	(62.7)	0.64
**Symptoms at Onset, n (%)**							
Initial GCS, median (IQR)	14	(8-15)	14	(8-15)	14	(8-15)	1
GCS on admission, median (IQR)	14	(3-15)	14	(3-15)	14	(3-15)	0.7
Decrease in vigilance	216	(63.0)	78	(62.4)	138	(63.3)	0.91
Loss of consciousness	160	(46.6)	58	(46.4)	102	(46.8)	1
WFNS grade, n (%)							
1	116	(33.8)	38	(30.4)	78	(35.8)	0.64
2	71	(20.7)	28	(22.4)	43	(19.7)	
3	13	(3.8)	3	(2.4)	10	(4.6)	
4	68	(19.8)	28	(22.4)	40	(18.3)	
5	75	(21.9)	28	(22.4)	47	(21.6)	
Headache	239	(69.7)	82	(65.6)	157	(72.0)	1
Acute onset headache	192	(56.0)	68	(54.4)	124	(56.9)	0.40
Not acute headache	47	(13.7)	13	(10.4)	34	(15.6)	0.40
Persistent headache	82	(23.9)	27	(21.6)	55	(25.2)	0.89
Duration of headache in days	4.13	(7.0)	2.91	(3.0)	4.75	(8.3)	0.12
Nuchal pain	53	(15.5)	18	(14.4)	35	(16.1)	1
Exercise-induced headache	24	(7.0)	13	(10.4)	11	(5.0)	**<0.05**
Sexual activity-induced headache	8	(2.3)	4	(3.2)	4	(1.8)	0.45
Defecation-induced headache	5	(1.5)	1	(0.8)	4	(1.8)	0.67
Meningism	54	(15.7)	14	(11.2)	40	(18.3)	0.27
Nausea/vomiting	188	(54.8)	65	(52.0)	123	(56.4)	0.49
Observed seizure	46	(13.4)	19	(15.2)	27	(12.4)	0.51
Possible seizure	108	(31.5)	39	(31.2)	69	(31.7)	1
Seizure *	148	(43.1)	55	(44.0)	93	(42.7)	0.82
Focal neurological deficit	73	(21.3)	21	(16.8)	52	(23.9)	0.25
Anisocoria	34	(9.9)	8	(6.4)	26	(11.9)	0.13
Pupils not reactive to light	29	(8.5)	9	(7.2)	20	(9.2)	0.56
Diplopia	12	(3.5)	5	(4.0)	7	(3.2)	0.54
Oculomotor disorder	16	(4.7)	5	(4.0)	11	(5.0)	1
Blurred vision	7	(2.0)	2	(1.6)	5	(2.3)	1
Vertigo	30	(8.7)	13	(10.4)	17	(7.8)	0.22
Confusion	87	(25.4)	31	(24.8)	56	(25.7)	0.67
Aphasia	18	(5.2)	6	(4.8)	12	(5.5)	1
Dysarthria	22	(6.4)	8	(6.4)	14	(6.4)	0.81
Hemisyndrome/motor deficit	38	(11.1)	12	(9.6)	26	(11.9)	0.85
Dys-/hyp-/paresthesia	10	(2.9)	4	(3.2)	6	(2.8)	0.74
Facial paresis	16	(4.7)	5	(4)	11	(5)	0.80
Neglect	2	(0.6)	1	(0.8)	1	(0.5)	1
Cardiac arrest	12	(3.5)	4	(3.2)	8	(3.7)	1
Hypertensive crisis **	57	(16.6)	20	(16)	37	(17)	0.88
Ear pressure/pain	2	(0.6)	0	(0)	2	(0.9)	0.55
Abdominal pain	7	(2)	2	(1.6)	5	(2.3)	1
Retrograde amnesia	17	(5)	6	(4.8)	11	(5)	1
Visual hallucinations	1	(0.3)	1	(0.8)	0	(0)	0.33
Behavioral change	4	(1.2)	3	(2.4)	1	(0.5)	0.11
Tinnitus	1	(0.3)	0	(0)	1	(0.5)	1

Abbreviations: n.a., not accessible; SD, standard deviation; CCI, Charlson Comorbidity Index; IQR, interquartile range; WLST, Withdrawal of life-supporting treatment; GOSE, Glasgow Outcome Scale-Extended; ICA, Internal Carotid Artery; MCA, Middle Cerebral Artery; AcomA, Anterior communicating Artery; ACA, Anterior Cerebral Artery; VA, Vertebral Artery; PCA, Posterior Cerebral Artery; PcomA, Posterior communicating Artery; BA, Basilar Artery; PICA, Posterior Inferior Cerebellar Artery; ICH, Intracerebral hemorrhage; SDH, Subdural hematoma; IVH, Intraventricular hemorrhage; WFNS, World Federation of Neurosurgery; GCS, Glasgow Coma Scale.

* Observed or suspected.

** Systolic blood pressure >200 mmHg.

### Sex-related analysis of symptom presentation

The median initial Glasgow Coma Scale (GCS) as well as the GCS on admission within all patients was 14 (interquartile range (IQR) 8-15), which did not differ between the two sexes. We provide a detailed sex-related list of all symptoms assessed at SAH onset in Table 1.

Among 46.6% (n=160) of all patients a loss of consciousness occurred during SAH onset, 43.1% (n=148) presented with an observed or suspected seizure and only 21.3% (n=73) with a focal neurological deficit on admission with a trend towards more women concerned (23.9%, n=52, vs. 16.8%, n=21; p=0.25). 69.7% (n=239) of all patients presented with headache and among these 56% (n=192) with acute onset headache and 23.9% (n=82) with persistent headache with a mean duration of 4.13 days (SD 7.0) without evidence for a sex-related difference. Exercise-induced headache was the only symptom with a statistically significant sex-related difference occurring more often within men (10.4%, n=13, vs. 5%, n=11; p=0.04).

### Subgroup analysis depending on Fisher scale

We additionally performed a subgroup analysis stratified by Fisher scale as a parameter of radiologically defined severity of aSAH. Patients were classified into two groups with Fisher scale 1-2 (n=37) and Fisher scale 3-4 (n=305). When analyzing the sex-related symptom presentation separately within these two groups, there was no evidence for a different clinical presentation between sexes at SAH onset.

### Subgroup analysis depending on WFNS grade

In a further subgroup analysis stratified by WFNS grade as a marker of clinical severity based on the GCS on admission, patients were classified into the groups WFNS 1-3 (n=200) defined as GCS 13-15 and WFNS 4-5 (n=143) defined as patients with a GCS ≤ 12. Within the subgroup of WFNS 1-3, men presented again more often with exercise-induced headache at SAH onset compared to women (18.8%, n=13, vs. 6.1%, n=8; p=0.007). In the subgroup of WFNS 3-4 with severely impaired consciousness, anisocoria revealed to occur statistically significantly more often among women (25.3%, n=22, vs. 10.7%, n=6; p=0.032), see Table 2.

**Table 2.** Subgroup analysis depending on aSAH severity.

	WFNS 1-3					WFNS 4-5				
	Male (n=69)		Female (n=131)		*p*	Male (n=56)		Female (n=87)		*p*
WFNS, n (%)										
1	n.a.	(n.a.)	n.a.	(n.a.)	n.a.	n.a.	(n.a.)	n.a.	(n.a.)	n.a.
2	n.a.	(n.a.)	n.a.	(n.a.)		n.a.	(n.a.)	n.a.	(n.a.)	
3	n.a.	(n.a.)	n.a.	(n.a.)		n.a.	(n.a.)	n.a.	(n.a.)	
4	n.a.	(n.a.)	n.a.	(n.a.)		n.a.	(n.a.)	n.a.	(n.a.)	
5	n.a.	(n.a.)	n.a.	(n.a.)		n.a.	(n.a.)	n.a.	(n.a.)	
Initial GCS median (IQR)	15	(14-15)	15	(14-15)	0.8	8	(3.3-12)	8	(4-12)	0.97
GCS on admission median (IQR)	15	(14-15)	15	(14-15)	0.86	3	(3-8)	3	(3-9)	0.19
Decrease in vigilance	27	(39.1)	54	(41.2)	0.88	51	(91.1)	84	(96.6)	0.26
Loss of consciousness	17	(24.6)	35	(26.7)	0.87	41	(73.2)	67	(77)	0.69
Headache	61	(88.4)	113	(86.3)	0.82	21	(37.5)	44	(50.6)	0.72
Acute onset headache	49	(71)	90	(68.7)	0.74	19	(33.9)	34	(39.1)	0.44
Not acute headache	11	(15.9)	23	(17.6)	0.85	2	(3.6)	11	(12.6)	0.2
Persistent headache	23	(33.3)	44	(33.6)	1	4	(7.1)	11	(12.6)	0.76
Duration of headache in days	3	(3.1)	4.61	(8.7)	0.24	2.4	(2.8)	5.29	(6.8)	0.38
Nuchal pain	11	(15.9)	30	(22.9)	0.28	7	(12.5)	5	(5.7)	0.05
Exercise-induced headache	13	(18.8)	8	(6.1)	**<0.01**	0	(0)	3	(3.4)	0.55
Sexual activity-induced headache	3	(4.3)	4	(3.1)	0.69	1	(1.8)	0	(0)	0.32
Defecation-induced headache	1	(1.4)	2	(1.5)	1	0	(0)	2	(2.3)	1
Meningism	11	(15.9)	35	(26.7)	0.21	3	(5.4)	5	(5.7)	1
Nausea/vomiting	45	(65.2)	81	(61.8)	0.65	20	(35.7)	42	(48.3)	0.16
Observed seizure	4	(5.8)	13	(9.9)	0.43	15	(26.8)	14	(16.1)	0.14
Possible seizure	14	(20.3)	22	(16.8)	0.57	25	(44.6)	47	(54)	0.31
Seizure (observed or suspected)	17	(24.6)	34	(26)	0.87	38	(67.9)	59	(67.8)	1
Focal neurological deficit	14	(20.3)	33	(25.2)	0.49	7	(12.5)	19	(21.8)	0.42
Anisocoria	2	(2.9)	4	(3.1)	1	6	(10.7)	22	(25.3)	**<0.05**
Pupils not reactive to light	0	(0)	1	(0.8)	1	9	(16.1)	19	(21.8)	0.52
Diplopia	4	(5.8)	7	(5.3)	1	1	(1.8)	0	(0)	0.3
Oculomotor disorder	2	(2.9)	9	(6.9)	0.34	3	(5.4)	2	(2.3)	0.31
Blurred vision	2	(2.9)	5	(3.8)	1	0	(0)	0	(0)	1
Vertigo	10	(14.5)	14	(10.7)	0.49	3	(5.4)	3	(3.4)	1
Confusion	25	(36.2)	41	(31.3)	0.53	6	10.7	15	(17.2)	1
Aphasia	3	(4.3)	6	(4.6)	1	3	(5.4)	6	(6.9)	1
Dysarthria	6	(8.7)	11	(8.4)	1	2	(3.6)	3	(3.4)	0.6
Hemisyndrome/motor deficit	6	(8.7)	12	(9.2)	1	6	(10.7)	14	(16.1)	1
Dys-/hyp-/paresthesia	4	(5.8)	3	(2.3)	0.23	0	(0)	3	(3.4)	0.54
Facial paresis	5	(7.2)	9	(6.9)	1	0	(0)	2	(2.3)	0.53
Neglect	1	(1.4)	1	(0.8)	1	0	(0)	0	(0)	1
Cardiac arrest	0	(0)	0	(0)	1	4	(7.1)	8	(9.2)	0.77
Hypertensive crisis	14	(20.3)	20	(15.3)	0.43	6	(10.7)	17	(19.5)	0.17
Ear pressure/pain	4	(5.8)	1	(0.8)	1	0	(0)	1	(1.1)	1
Abdominal pain	1	(1.4)	4	(3.1)	0.66	1	(1.8)	1	(1.1)	1
Retrograde amnesia	6	(8.7)	8	(6.1)	0.56	0	(0)	3	(3.4)	0.54
Visual hallucinations	1	(1.4)	5	(3.8)	0.34	0	(0)	0	(0)	1
Behavioral change	3	(4.3)	1	(0.8)	0.12	0	(0)	0	(0)	1
Tinnitus	4	(5.8)	1	(0.8)	1	0	(0)	0	(0)	1

### Subgroup analysis depending on aneurysm location

When analyzing the subgroups of anterior (n=271) and posterior circulation aneurysms (n=72), there was no evidence for sex-related differences in symptom presentation at SAH onset. Only meningism tended to occur more often in women with aneurysms of the anterior circulation, but without significance (18.9%, n=32, vs. 8.8%, n=9; p=0.064), see Table 3.

**Table 3.** Subgroup analysis depending on aneurysm location.

	Anterior circulation aneurysm					Posterior circulation aneurysm				
	Male (n=102)		Female (n=169)		*p*	Male (n=23)		Female (n=49)		*p*
WFNS, n (%)										
1	33	(32.4)	65	(38.5)	0.67	5	(21.7)	13	(26.5)	0.5
2	20	(19.6)	35	(20.7)		8	(34.8)	8	(16.3)	
3	2	(2)	6	(3.6)		1	(4.3)	4	(8.2)	
4	23	(22.5)	30	(17.8)		5	(21.7)	10	(20.4)	
5	24	(23.5)	33	(19.5)		4	(17.4)	14	(28.6)	
Initial GCS median (IQR)	14	(8-15)	14	(8.5-15)	0.99	14	(8-15)	13	(6.5-15)	0.92
GCS on admission median (IQR)	13.5	(3-15)	14	(5-15)	0.67	14	(7-15)	13	(3-15)	0.57
Decrease in vigilance	63	(61.8)	106	(62.7)	0.9	15	(65.2)	32	(65.3)	1
Loss of consciousness	49	(48)	77	(45.6)	0.71	9	(39.1)	25	(51)	0.45
Headache	66	(64.7)	119	(70.4)	0.53	16	(69.6)	38	(77.6)	0.32
Acute onset headache	55	(53.9)	94	(55.6)	0.35	13	(56.5)	30	(61.2)	1
Not acute headache	11	(10.8)	25	(14.8)	0.7	2	(8.7)	9	(18.4)	0.48
Persistent headache	21	(28.4)	42	(24.9)	0.88	6	(26.1)	13	(26.5)	1
Duration of headache in days	3	(3.2)	4.9	(9.2)		2.4	(2.1)	4.1	(3.2)	
Nuchal pain	11	(10.8)	28	(16.6)	0.46	7	(30.4)	7	(14.3)	0.11
Exercise-induced headache	9	(8.8)	8	(4.7)	0.11	4	(17.4)	3	(6.1)	0.2
Sexual activity-induced headache	3	(2.9)	4	(2.4)	0.69	1	(4.3)	0	(0)	0.32
Defecation-induced headache	1	(1)	4	(2.4)	0.66	0	(0)	0	(0)	1
Meningism	9	(8.8)	32	(18.9)	0.06	5	(21.7)	7	(16.3)	0.16
Nausea/vomiting	56	(54.9)	97	(57.4)	0.8	9	(39.1)	26	(53.1)	0.31
Observed seizure	17	(16.7)	23	(13.6)	0.49	2	(8.7)	4	(8.2)	1
Possible seizure	36	(35.3)	52	(30.8)	0.5	3	(13)	17	(34.7)	0.09
Seizure (observed or suspected)	50	(48)	72	(42.6)	0.32	6	(26.1)	21	(42.9)	0.12
Focal neurological deficit	15	(14.7)	43	(25.4)	0.11	1	(4.3)	9	(18.4)	0.51
Anisocoria	7	(6.9)	18	(10.7)	0.39	1	(4.3)	8	(16.3)	0.25
Pupils not reactive to light	7	(6.9)	14	(8.3)	0.82	2	(8.7)	6	(12.2)	1
Diplopia	2	(2)	4	(2.4)	1	3	(13)	3	(6.1)	0.38
Oculomotor disorder	4	(3.9)	8	(4.7)	1	1	(4.3)	3	(6.1)	1
Blurred vision	1	(1)	4	(2.4)	1	1	(4.3)	1	(2)	1
Vertigo	9	(8.8)	13	(7.7)	0.47	4	(17.4)	4	(8.2)	0.41
Confusion	25	(24.5)	46	(27.2)	0.75	6	(26.1)	10	(20.4)	0.75
Aphasia	6	(5.9)	10	(5.9)	0.79	0	(0)	2	(4.1)	1
Dysarthria	6	(5.9)	11	(6.5)	1	2	(8.7)	3	(6.1)	0.64
Hemisyndrome/motor deficit	12	(11.8)	23	(13.6)	1	0	(0)	3	(6.1)	0.54
Dys-/hyp-/paresthesia	4	(3.9)	4	(2.4)	0.44	0	(0)	2	(4.1)	0.54
Facial paresis	3	(2.9)	10	(5.9)	0.55	2	(8.7)	1	(2)	0.25
Neglect	1	(1)	1	(0.6)	1	0	(0)	0	(0)	1
Cardiac arrest	0	(0)	4	(2.4)	0.3	4	(17.4)	4	(8.2)	0.26
Hypertensive crisis	16	(15.7)	24	14.2	0.73	4	(17.4)	13	(26.5)	0.55
Ear pressure/pain	0	(0)	1	(0.6)	1	0	(0)	1	(2)	1
Abdominal pain	2	(2)	3	(1.8)	1	0	(0)	2	(4.1)	1
Retrograde amnesia	4	(3.9)	9	(5.3)	1	2	(8.7)	2	(4.1)	0.59
Visual hallucinations	1	(1)	0	(0)	0.33	0	(0)	0	(0)	1
Behavioral change	2	(2)	1	(0.6)	0.26	1	(4.3)	0	(0)	0.33
Tinnitus	0	(0)	1	(0.6)	1	0	(0)	0	(0)	1

## Discussion

Patients with aSAH represent a severely ill patient group with often devastating outcome. ^17^^,^^18^ Earliest possible detection and treatment of the ruptured aneurysm is imperative for the improvement of outcome. ^18^ In patients with aSAH, differences in clinical and subjective symptoms at onset between both sexes have not been studied in-depth so far with only few studies addressing symptoms of aSAH patients in general without differentiating between sexes. ^19^ Our primary hypothesis was that similarly to the findings in cardiovascular disease, symptom presentation in female aSAH patients might also differ.

In patients with MI, women often present with atypical symptoms as dizziness, nausea or extracardiac pain at onset compared to men resulting in lower rates of detection, treatment and worsened outcome in women. ^5^^–^^8^^,^^12^

In our study population, we found that only exercise-induced headache was more frequently reported by men compared to women. For all other clinical symptoms at aSAH onset, there was no evidence for differentiating symptoms with respect to sex. To reduce the bias that symptoms could not be assessed correctly due to limitations to get the medical history in patients with impaired consciousness, we conducted a subgroup analysis considering patients with only slight to modest decline in consciousness (WFNS 1-3). In this subgroup, the findings did not change thereby supporting and validating in a first step our results from the whole study group. Regarding patients with a severe decline in consciousness, only anisocoria occurred more frequently within female patients (WFNS 4-5; p=0.032). In line with that, ruptured Pcom aneurysms were found more frequently in women (p=0.005), with this type of aneurysm representing a common cause of acute oculomotor nerve palsy resulting in anisocoria. ^20^ Accordingly, women with acute onset anisocoria should be screened even more carefully for an underlying ruptured PcomA aneurysm.

In our study, we detected only few differing symptoms at onset with respect to sex in aSAH patients compared to patients with MI, which might be explained by the fact that the epicardial innervation is dermatoma-specific enabling a vast range of different, atypical or extracardiac symptoms. In contrast, the intracranial innervation is not, thus symptoms in aSAH patients are mainly stereotype with headache (69.7%), nausea or vomiting (54.8%) and decrease of consciousness (63%) when intracranial pressure rises due to local or generalized space-occupying effects or acute hydrocephalus after aSAH or consist of focal neurological deficits depending on the aneurysm location.

In previous aSAH patient cohorts, women were more often affected compared to men, ^2^^,^^9^ which is in line with our findings. Humoral factors as decreased estrogen levels after menopause contribute to an increased risk of aneurysm formation in older females. ^21^^,^^22^ Interestingly, female sex has been associated with an increased risk of aneurysm formation, but not with an increased risk of aneurysm rupture. ^23^ Aneurysm location has been reported to differ between both sexes with more ruptured aneurysms of the ACA in men, ^11^ which could be confirmed in our study with predominantly ruptured AcomA aneurysm in men. In women the ICA has been described as the most affected vessel of ruptured aneurysms, ^11^ whereas we detected most frequently MCA aneurysms in women. However, PcomA aneurysms were found significantly more often in women compared to men.

The strength of our study is that we provide a broad overview of possible clinical symptoms in aSAH patients within a large and homogenous patient cohort ( Table 1), thereby offering a clinical and practice-oriented guide with the aim of rapid and accurate diagnosis and treatment. We also performed a subgroup analysis taking into account the Fisher scale, WFNS grade and aneurysm location to rule out a possible bias due to limited assessment of symptoms at onset in patients with impaired consciousness (WFNS 4-5). Limitations of the study are firstly the single-center and retrospective type of study possibly leading to a certain bias in comparability and generalization of study results. Furthermore, a validation cohort with larger patient numbers should be investigated to confirm our results. In addition, due to the high amount of decreased vigilance (63%) in aSAH patients, the case history often had to be taken by a third party, which might result in a certain inaccuracy of the assessed symptoms at onset. To rule out that bias, we performed a subgroup analysis adjusted for the severity of hemorrhage (Fisher scale) and the level of consciousness (WFNS grade), respectively. By demonstrating that there was again no evidence for gender-related differing symptoms in patients with only slight to moderate decrease in vigilance (WFNS 1-3), the results of the whole study group were confirmed.

In conclusion, our results show no evidence for relevant sex-related differences in symptom presentation at onset in aSAH patients with stable findings when only analyzing patients with slight to moderate decrease in consciousness (WFNS 1-3). However, due to smaller patient numbers in subgroup analyses, the absence of evidence for relevant sex-related differences in symptom presentation in patients with aSAH does not imply that there are no such differences. Further studies with comparative larger patient cohorts preferably with aSAH, but also other acute intracranial processes as hemorrhagic or ischemic stroke are needed to verify and confirm these results and provide a better understanding of possible sex related differences and specific treatment options in these patients.

## Author contributions

Laura P. Westphal conducted data processing and interpretation and wrote the first draft of the manuscript. Stefan Y. Bögli was involved in data collection and performed the statistical analysis. Jana Werner and Francesca Casagrande were involved in data collection and critically revised the manuscript. Emanuela Keller critically revised the manuscript. Giovanna Brandi designed the study, was involved in data analysis and critically revised the manuscript. All authors contributed to manuscript revision and approved the final version of the manuscript.

## Data availability

The original data set can be found on Zenodo. Sex-related differences in symptom presentation of patients with aneurysmal subarachnoid hemorrhage. DOI: https://doi.org/10.5281/zenodo.7007417. ^24^

This project contains the following underlying data:

- Retrospective study regarding sex-related differences in symptom presentation of patients with aneurysmal subarachnoid hemorrhage.

Data are available under the terms of the Creative Commons Attribution 4.0 International license (CC-BY 4.0).

### Reporting guidelines

The study was reported according to the STROBE guidelines for observational studies. ^25^

Zenodo. Completed STROBE guidelines checklist for observational studies of the manuscript “Sex-related differences in symptom presentation of patients with aneurysmal subarachnoid hemorrhage”. DOI: https://doi.org/10.5281/zenodo.7102315. ^26^
