# Supplementary material for: Sex-related differences in symptom presentation of patients with aneurysmal subarachnoid hemorrhage
Source: F1000Res. 2023 Mar 13;11:1149. Originally published 2022 Oct 7. [Version 2] doi: 10.12688/f1000research.124123.2 (PMC10354455; doi:10.12688/f1000research.124123.2)
Supplement: Peer review history

# Peer review: Reviewer response for version 2

**DOI:** 10.5256/f1000research.145418.r179289

**Published:** 2023-07-18

**Authors:** Sabah Rehman

**Affiliations:** 1 University of Tasmania, Hobart, Tasmania, Australia

The authors have comprehensively observed sex differences in the clinical symptoms of patients with aSAH. The found exercise induced headache to be more common in men than women and anisocoria more frequently in women than men. However they found no evidence based sex related differences in symptom presentation at aSAH onset. The authors have done good job on this. Some details in paragraph 2 of discussion could be added about any clinical evidence of noted sex differences in their study (e.g., anisocoria) with the outcomes (e.g., morbidity or delays in management).This could make somewhat clear that why examining sex related differences in symptoms is important besides being helpful with an early diagnosis. I leave it to authors if they want to add this to discussion. I don't have further comments.

Is the work clearly and accurately presented and does it cite the current literature?

Yes

If applicable, is the statistical analysis and its interpretation appropriate?

Yes

Are all the source data underlying the results available to ensure full reproducibility?

Yes

Is the study design appropriate and is the work technically sound?

Yes

Are the conclusions drawn adequately supported by the results?

Yes

Are sufficient details of methods and analysis provided to allow replication by others?

Yes

Reviewer Expertise:

stroke epidemiology and clinical research

I confirm that I have read this submission and believe that I have an appropriate level of expertise to confirm that it is of an acceptable scientific standard.

# Peer review: Reviewer response for version 2

**DOI:** 10.5256/f1000research.145418.r166248

**Published:** 2023-05-16

**Authors:** Matthias Haenggi

**Affiliations:** 1 Department of Intensive Care Medicine, Bern University Hospital, Inselspital, University of Bern, Bern, Switzerland

I have no further comments, thank you.

Is the work clearly and accurately presented and does it cite the current literature?

Yes

If applicable, is the statistical analysis and its interpretation appropriate?

Yes

Are all the source data underlying the results available to ensure full reproducibility?

Yes

Is the study design appropriate and is the work technically sound?

Yes

Are the conclusions drawn adequately supported by the results?

Yes

Are sufficient details of methods and analysis provided to allow replication by others?

Yes

Reviewer Expertise:

Critical care medicine

I confirm that I have read this submission and believe that I have an appropriate level of expertise to confirm that it is of an acceptable scientific standard.

# Peer review: Reviewer response for version 1

**DOI:** 10.5256/f1000research.136295.r162644

**Published:** 2023-03-07

**Authors:** Matthias Haenggi

**Affiliations:** 1 Department of Intensive Care Medicine, Bern University Hospital, Inselspital, University of Bern, Bern, Switzerland

Dr Westphal and her colleagues aimed to answer the question if there are any differences in symptom presentation for aSAH with regard to sex, similar to different symptom presentation between women and men with ACS/MI. Although there are few distinctions in baseline characteristics and aneurysm location, symptoms at onset of SAH did not differ between women and men. This holds true after adjusting for severity and aneurysm location.

 The manuscript is clear, concise and well-written/easy to understand. The conclusions drawn are based on the results. The limits are outlined, but I am missing one or two sentences about the troubles frequently occurring if trial results are “neutral” or “negative”: for theoretical reasons, failure to prove a difference does not imply that there is no difference (“absence of evidence is not evidence of absence”). It can simply occur if the number of events is low, as it has happened here in subgroup analysis, or the total n are low. It is the clinical context which is important, not the p-value. Therefore I am not convinced that “ *a validation cohort with larger patient numbers should be investigated to confirm our results*”, as stated by the authors. Variation of symptoms in presentation is high, so knowledge of a statistically significant sex difference in a symptom cannot rule in/rule out aSAH.

 There are some minor issues:

- Fisher score WFNS grade/GOSE: for the readers who do not remember the scoring systems, please present in a few words the grading (the higher the score, the worse…)
- Table 1: " *WLST*" is not defined, I assume 'withdrawal of life support therapies'

Is the work clearly and accurately presented and does it cite the current literature?

Yes

If applicable, is the statistical analysis and its interpretation appropriate?

Yes

Are all the source data underlying the results available to ensure full reproducibility?

Yes

Is the study design appropriate and is the work technically sound?

Yes

Are the conclusions drawn adequately supported by the results?

Yes

Are sufficient details of methods and analysis provided to allow replication by others?

Yes

Reviewer Expertise:

Critical care medicine

I confirm that I have read this submission and believe that I have an appropriate level of expertise to confirm that it is of an acceptable scientific standard, however I have significant reservations, as outlined above.

# Response

**Published:** 2023-03-09

**Authors:** Laura Westphal

**Affiliations:** University Hospital Zurich, Switzerland

We thank the reviewer for the precise and valuable review of the article. We have now completed an updated version of the manuscript, in which we adapted and included the suggested points according to the review.

 In detail, we included in the conclusion part at the end of the discussion that, due to smaller patient numbers in subgroup analyses, the absence of evidence for relevant sex-related differences in symptom presentation of patients with aSAH does not imply that there are no such differences. We additionally adapted the sentence that our results need to be validated in the way that results need to be verified and confirmed in further studies in the future with larger patient cohorts.

 Furthermore, we thank the reviewer for the important aspect to explain in more detail the Fisher, WFNS and GOS-E scale used for clinical and diagnostic classification of patients with aSAH. The according changes can be found in the methods section. 

 Additionally, we added a list of abbreviations at the end of table 1 in order to explain all abbreviations used in the table, including WLST for "Withdrawal of life-supporting treatment".

 We hope that we could address all mentioned points of the review within the adapted manuscript, which is now available on the website.
